# Unraveling the symmetry of Al_5_C_3_N

**DOI:** 10.1107/S2052520626002544

**Published:** 2026-04-22

**Authors:** Vitalii Shtender, Chin Shen Ong, Pedro Berastegui, Olivier Donzel-Gargand, Johan Cedervall, Charles Hervoches, Premek Beran, Olle Eriksson, Ulf Jansson

**Affiliations:** a Department of Chemistry, Ångström Laboratory Uppsala University Box 523 Uppsala S-75120 Sweden; b Department of Physics and Astronomy Uppsala University Uppsala S-75120 Sweden; c Division of Solar Cell Technology, Department of Materials Science and Engineering Uppsala University Uppsala S-75121 Sweden; d Nuclear Physics Institute CAS Rez 25068 Czechia; e European Spallation Source, ESS ERIC, Lund, S-221 00, Sweden; f WISE Wallenberg Initiative Materials Science Uppsala University Uppsala Sweden; University of Geneva, Switzerland

**Keywords:** aluminium carbonitride, crystal structure, synthesis, electronic structure

## Abstract

High-temperature ceramic compound Al_5_C_3_N belongs to the scarcely studied Al–C–N system. It was originally reported as an ordered compound in noncentrosymmetric space group *P*6_3_*mc* and described as a nanolaminate with an –Al_2_C–AlN–Al_2_C_2_– stacking sequence. The recently reported structural disorder in the related compound Al_4_SiC_4_ led us to question this proposed structure for Al_5_C_3_N and investigate the possibility of a disordered structure in the centrosymmetric space group *P*6_3_/*mmc*. In the present work, we employed different synthesis routes to maximize the yield and quality of the desired phase, and applied a variety of techniques to probe the Al_5_C_3_N crystal structure. Our single-crystal X-ray diffraction analysis clearly indicates that noncentrosymmetric space group *P*6_3_*mc* must be rejected. From a joint refinement of single-crystal X-ray and neutron powder diffraction data, the occupancies of C and N were refined at two sites in *P*6_3_/*mmc* resulting in the stacking sequence –Al_2_C–Al(C/N)–Al_2_(C/N)_2_–. Furthermore, DFT calculations show that a centrosymmetric disordered structure described in a supercell has the lowest energy, 0.2 eV per formula unit, relative to the previously reported *P*6_3_*mc* structure. The calculated band structure shows both direct and indirect bandgaps, which leads to implications for the physical properties. Finally, STEM analysis provides additional evidence that the crystal structure of Al_5_C_3_N is better described in centrosymmetric space group *P*6_3_/*mmc*.

## Abstract 2

From experimental and theoretical techniques employed in this thorough reexamination of the structure of Al_5_C_3_N, the crystal structure is better described in centrosymmetric space group *P*6_3_/*mmc* (No. 194) rather than in the previously reported noncentrosymmetric space group *P*6_3_*mc* (No. 186).

## 1. Introduction

**Figure 1:**
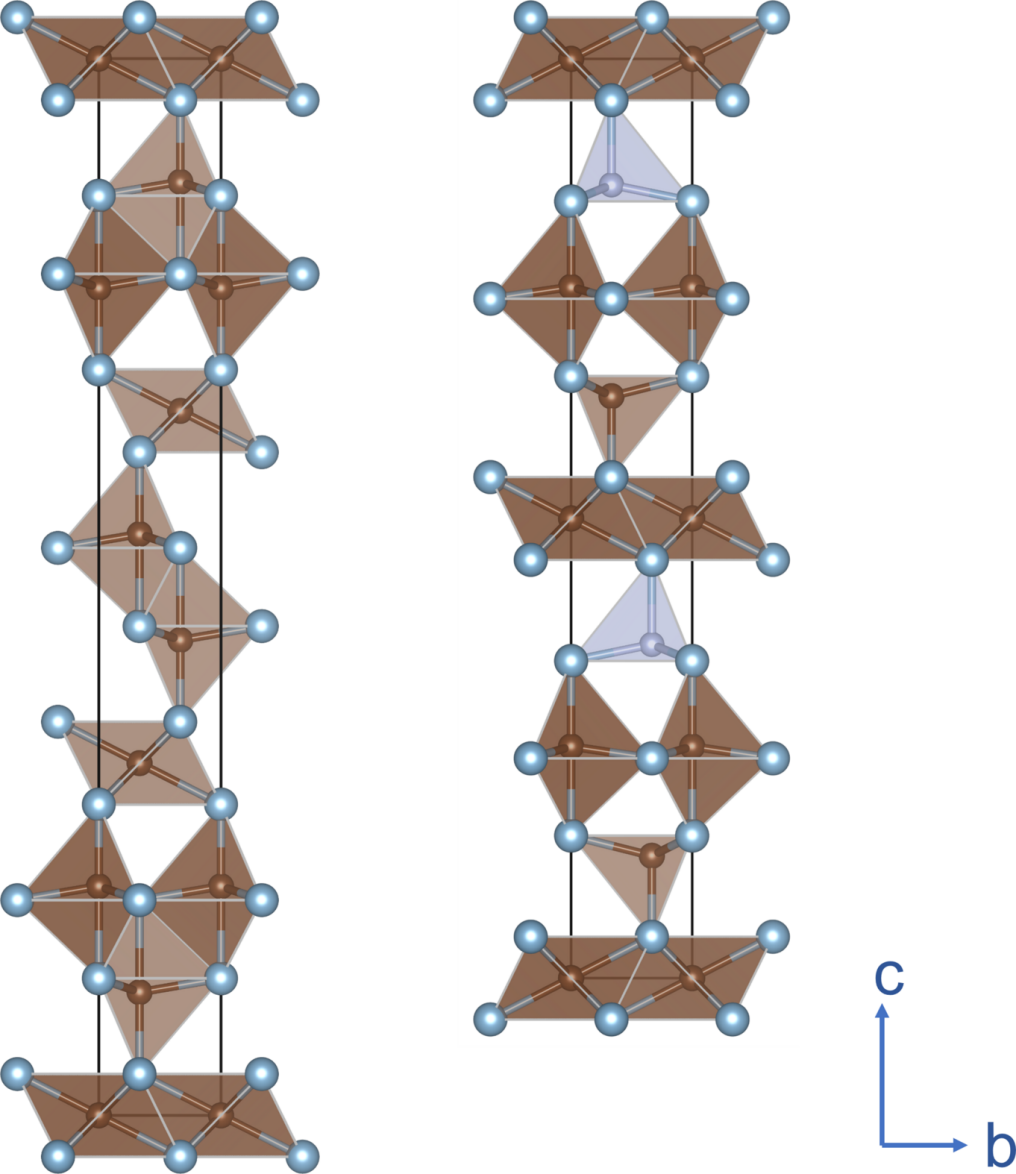
Projection of the crystal structures of Al_4_C_3_ (Gesing & Jeitschko, 1995 ▸) (left) showing the fivefold coordination of C (brown) in –Al_2_C_2_– blocks between the –Al_2_C– blocks with C in sixfold coordination to Al (blue) atoms; Al_5_C_3_N (right) as reported by Jeffrey & Wu (1963 ▸) with additional –AlN– layers (gray tetrahedra).

Several ternary phases have been described in the Al–C–N system. The hexagonal phase of Al_5_C_3_N was first reported by von Stackelberg *et al.* (1935 ▸), von Stackelberg & Spiess (1935 ▸). Jeffrey & Wu (1963 ▸) investigated the crystal structure of small crystals obtained from the walls of a carbon crucible after heating AlN powder to about 2273 K in a nitro­gen atmosphere. They observed a series of phases with a chimney-like structure having the general formula Al_4+*n*_C_3_N*_n_* with *n* = 1–4. The aluminium carbonitrides with an odd *n* number, Al_5_C_3_N and Al_7_C_3_N_3_, were described with a hexagonal structure in space group *P*6_3_*mc* (#186), while the members with even *n* number, Al_6_C_3_N_2_ and Al_8_C_3_N_4_, were described as rhombohedral in space group *R*3*m* (#166). Based on the single-crystal structure analyses of Jeffrey & Wu (1963 ▸) and Jeffrey & Wu (1966 ▸), the structure of Al_5_C_3_N can be described as a nanolaminate with AlN layers between Al_2_C_2_ and Al_2_C blocks, whereas in Al_8_C_3_N_4_, for example, the repeating sequence is –Al_2_C_2_–AlN–AlN–Al_2_C–AlN–AlN–. Thus, the Al_4+*n*_C_3_N*_n_* phases could be described as a series of compounds with the end members Al_4_C_3_ (for *n* = 0) and theoretically AlN (for *n* = ∞). The ordered structure of Al_5_C_3_N can also be visualized with layers of polyhedra with C in either tetrahedral or trigonal bipyramidal, and octahedral coordination (as in Al_4_C_3_) and N in tetrahedral coordination, as shown in Fig. 1 ▸.

The stability of Al_5_C_3_N has been confirmed experimentally (Oden & McCune, 1990 ▸; Schneider *et al.*, 1979 ▸; Pietzka & Schuster, 1996 ▸) and according to a calculated phase diagram, this phase melts incongruently at 2526 K (Qiu & Metselaar, 1997 ▸). Samples with the Al_5_C_3_N phase have been prepared from mixtures of Al_4_C_3_ and AlN by hot pressing in inert atmospheres (Oden & McCune, 1990 ▸; Schneider *et al.*, 1979 ▸) or heating in an H_2_ atmosphere (Pietzka & Schuster, 1996 ▸), by plasma jet cladding (Mu *et al.*, 2011 ▸), and by heating Al_4_C_3_ in an N_2_ atmosphere (von Stackelberg *et al.*, 1935 ▸). However, the existence of pure aluminium carbonitrides with *n* > 1 has been difficult to reproduce, and several authors have suggested that they are stabilized by impurities (Pietzka & Schuster, 1996 ▸; Qiu & Metselaar, 1997 ▸).

Since other elements such as Si may play a role in stabilizing the Al_4+*n*_C_3_N*_n_* phases with *n* > 1, it is interesting to compare the Al–C–N system with the Al–C–Si system, where ternary phases with the general formula Al_4_Si*_n_*C_3+*n*_ have been observed. First reported in 1961, the hexagonal unit cell of Al_4_SiC_4_ is very similar to that of Al_5_C_3_N (Schneider *et al.*, 1979 ▸; Barczak, 1961 ▸). We have recently revisited the crystal structure of Al_4_SiC_4_ using a combination of experimental and theoretical methods and present a crystal structure with a mixed occupancy of Si and Al and the presence of Al vacancies, in contrast to the previously proposed ordered structure, which might have some interesting implications for the properties of Al_4_SiC_4_ (Ong *et al.*, 2024 ▸).

Many applications have been proposed for phases in the Al–C–Si system, and it can be assumed that similar properties are also exhibited by the phases in the Al–C–N system. However, only one theoretical study of the electronic structure of Al_5_C_3_N has been published, indicating that this phase is a narrow bandgap semiconductor (Xu *et al.*, 2011 ▸). Furthermore, a mainly covalent–ionic bonding with stronger Al—N bonds than Al—C bonds was predicted, consistent with a nanolaminated structure and possibly higher ductility than most MAX phases and Al_4_SiC_4_ (Liao *et al.*, 2006 ▸)

A number of discrepancies in the structural determination of Al_5_C_3_N were reported and attributed to both experimental errors and stoichiometric defects (Jeffrey & Wu, 1963 ▸). Subsequent studies on this phase have used the original determination in noncentrosymmetric space group *P*6_3_*mc*. However, the same extinction conditions are observed in the centrosymmetric space group *P*6_3_/*mmc* for a disordered structure, and the original structure proposed by Jeffrey & Wu (1963 ▸) may be incorrect. The aim of this work is therefore to make a reassessment of this structure with a more detailed and complete study using both experimental and theoretical methods. We have used single-crystal X-ray diffraction and neutron powder diffraction to study the structure of Al_5_C_3_N. Furthermore, we have used scanning transmission electron microscopy (STEM) to gain additional information, and density functional theory (DFT) calculations have been used to determine the most stable structural configuration of this phase.

## 2. Methods

Al_5_C_3_N was synthesized from Al_4_C_3_ (Alfa Aesar 99+%) as powder packed in a cylindrical graphite crucible with 40 mm internal diameter. The crucible was placed in the chamber of a graphite furnace from Thermal Technology LLC that was evacuated and refilled with Ar (6N) three times before N_2_ (g) was mixed using mass flow controllers to obtain a 1% N_2_/Ar atmosphere. After allowing the atmosphere inside the chamber to stabilize, the chamber was heated to 2223 K at a rate of 25 K min^−1^ and kept at that temperature for 60 min. The cooling rate was set at 75 K min^−1^, but the furnace cooled naturally below approximately 1273 K. The partially sintered sample was covered with a thin layer of graphitic carbon that was scraped off before the rest of the sample was removed, ground and packed in the graphite crucible. The heat treatment and this process were repeated five times, after which no further reaction of the remaining Al_4_C_3_ could be measured.

A small single crystal of Al_5_C_3_N was measured using a Bruker D8 single-crystal X-ray diffractometer with Mo *K*α radiation (λ = 0.71073 Å). The diffractometer was equipped with an Incoatec Microfocus Source (IμS) and an APEX II CCD area detector. Single-crystal X-ray diffraction (SCXRD) data reduction and numerical absorption corrections were performed using the *APEX3* software from Bruker (2015 ▸). Structure solution using the *SUPERFLIP* method and subsequent refinement were carried out in *JANA2020* (Petříček *et al.*, 2023 ▸).

Given the near identical atomic form factor of C and N for X-ray radiation (at *s* = 0, C: 6e^−^; N: 7e^−^), but significantly different for neutrons (scattering length, C: 6.64 fm; N: 9.36 fm), a neutron powder diffraction measurement was performed using the MEREDIT diffractometer at the Nuclear Physics Institute CAS in Rez, Czech Republic. A neutron beam with a wavelength of 1.46 Å was applied using a copper mosaic monochromator (reflection 220). A diffraction pattern in a 2θ range of 4–144° with steps of 0.08° was collected at room temperature. The acquired powder diffraction patterns were analyzed with the software *FullProf* (Rodríguez-Carvajal, 2001 ▸) using the Rietveld method.

Powder morphology and composition were studied using a ZEISS Leo 1550 field emission scanning electron microscope (SEM) equipped with an Oxford X-max detector (80 mm^2^) for the energy-dispersive X-ray spectroscopy (EDS). Raman spectra were collected with a Renishaw inVia confocal Raman microscope using a 532 nm laser. IR spectra were collected using a Perkin Elmer Spectrum One instrument, equipped with a KBr/PE beam-splitter, DTGS/KBr detector and a Pike GladiATR diamond ATR unit, at a resolution of 2 cm^−1^. The polycrystalline samples were also studied by powder X-ray diffraction (PXRD) in θ–2θ mode using Cu *K*α radiation. The measurements were done with a Bruker D8 Advance instrument equipped with a Lynxeye-XE detector and Ni filter. Quantitative phase analysis was done using the reference intensity ratio method (Hubbard & Snyder, 1988 ▸). The results from these measurements are presented in the supporting information.

DFT calculations were performed using *Quantum ESPRESSO* (Giannozzi *et al.*, 2009 ▸), which uses a plane-wave basis set. The plane-wave cut-off for the DFT calculation was set to 85 Ry for the plane-wave expansion of the wavefunctions using the scalar-relativistic optimized norm-conserving Vanderbilt pseudopotential (Hamann, 2013 ▸) obtained from the PSEUDODOJO project (van Setten *et al.*, 2018 ▸). The Perdew–Burke–Ernzerhof functional within generalized gradient approximations was used as the DFT exchange-correlation functional. For all structures, all components of all forces were minimized within the convergence threshold of 10^−5^ Ry per Bohr radius, and the total energy was also minimized within the convergence threshold of 10^−8^ Ry. Integrations over reciprocal space were performed on 15×15×2 and 8×8×2 **k**-grids for the unit cell and (2×2)-supercell, respectively.

Al_5_C_3_N crystals were also investigated with transmission electron microscopy (TEM). The lamellae were prepared using a Ga-based focused ion beam CrossBeam550 from Zeiss. The ion acceleration voltage was gradually reduced to 1 kV to minimize the polishing damage in the final lamella. A particular effort was made to include the *c* axis of the crystal in the plane of the lamella. The TEM analyses were performed at 200 kV on a Titan Themis 200 microscope [Thermofisher (formerly FEI)] equipped with a Cs probe-corrector and a SuperX EDS system. The sample was loaded the day before the TEM study for improved stability. In Scanning (S)TEM, the high-angle annular dark field (HAADF) detector and the annular bright field detector collected signals ranging between 70 and 200 mrad and 10–25 mrad, respectively. The simulated STEM images were calculated with a multi-slice approach using *Dr. Probe* (Barthel, 2018 ▸) software with the unit cell refined from neutron diffraction data as input.

## 3. Results and discussion

### 3.1. Synthesis of Al_5_C_3_N

Al_4_C_3_ is commonly used as a reactant in the preparation of MAX phases (Gauthier-Brunet *et al.*, 2009 ▸) and has been studied as a constituent in multicomponent systems and Al alloys (Tham *et al.*, 2001 ▸; Ci *et al.*, 2006 ▸). However, it is easily hydrolyzed (Nýblová *et al.*, 2018 ▸) and can be detrimental to the mechanical properties if present as an impurity. Al_4_C_3_ decomposes at 2423 K into graphite and a liquid phase of C dissolved in Al (Deffrennes *et al.*, 2019 ▸), but a significant volatility of Al has been observed during annealing at temperatures above 1973 K (Chupka *et al.*, 1958 ▸; Plante & Schreyer, 1966 ▸; Li *et al.*, 2011 ▸). The related Al_5_C_3_N phase, on the other hand, is stable in ambient air and on cooling from high temperature, but the synthesis of this compound is not as straightforward. In the literature, two different routes have been used to synthesize Al_5_C_3_N: (i) heating of Al_4_C_3_ in an inert atmosphere with N_2_ and (ii) a high-temperature reaction between Al_4_C_3_ and AlN. Route (i) was used by von Stackelberg *et al.* in the 1930s by heating Al_4_C_3_ in an atmosphere of N_2_ diluted in H_2_ at temperatures below 2473 K (von Stackelberg *et al.*, 1935 ▸). Al_5_C_3_N can be considered to be an intermediate phase in the nitridation of Al_4_C_3_ to AlN, and high temperatures or partial pressures of N_2_ will increase the amount of AlN that forms. Their observations also suggested that the reaction started with a partial delamination of Al_4_C_3_ and sublimation before forming the carbonitride. Route (ii) has been used with the sintering of mixtures of Al_4_C_3_ and AlN at atmospheric pressure (Pietzka & Schuster, 1996 ▸) or by hot-pressing (Schneider *et al.*, 1979 ▸) at 2073 K for 30–60 min. However, the qualities of these samples are difficult to assess as no diffraction patterns were published. Reacting Al_4_C_3_ with AlN as a nitro­gen source in inert atmospheres in route (ii) leads to the formation of the carbonitride at lower temperatures compared to route (i), and the reaction is apparently fast. However, Al_5_C_3_N reacts with AlN to form Al_6_C_3_N_2_, and impurities in the reactants or the atmosphere may result in the formation of an aluminium oxycarbonitride (Oden & McCune, 1990 ▸; Inuzuka *et al.*, 2010 ▸).

In our study, we have investigated both routes (i) and (ii) to determine the most efficient way to synthesize Al_5_C_3_N. Table 1 ▸ summarizes the results of phase analyses of Al_4_C_3_ samples that have been heated in different conditions using route (i), *i.e.* heating of Al_4_C_3_ in an N_2_ atmosphere. All samples synthesized by this reaction are covered with a layer of graphitic carbon that can be mechanically removed, and the carbon content has thus not been considered. After heating in an inert Ar atmosphere (samples 1 and 2), no other phases are observed, but a weight loss of about 9% at 2173 K and 16% at 2273 K was measured due to the decomposition of Al_4_C_3_ and evaporation of Al. The Al_5_C_3_N phase was observed after heating Al_4_C_3_ in atmospheres with N_2_, and longer annealing times at lower N_2_ partial pressures increased the fraction of the carbonitride phases (samples 3 to 8). Moreover, two AlN phases with the same crystal structure (wurtzite) but with different unit-cell volumes, 41.75 and 42.19 Å^3^, were found in all samples heated in an atmosphere with > 1.5% N_2_ in Ar. The smaller cell is consistent with the reported unit cell for AlN and forms from the decomposition of Al_5_C_3_N (sample 6), while the phase with the larger unit cell should form due to the nitridation of Al in nitro­gen-rich atmospheres. The reason for this difference in unit-cell volume is unclear. In summary, samples with Al_5_C_3_N as the main phase (> 50%) were obtained from the nitridation of Al_4_C_3_ in atmospheres with low partial pressures (1–1.5%) of N_2_ and temperatures in the range 2223–2273 K. SEM images of the Al_5_C_3_N crystals together with EDX maps, were collected (Figs. S1 and S2, respectively).

We have also investigated the formation of Al_5_C_3_N using route (ii), *i.e.* heating of mixtures of Al_4_C_3_ and AlN in different ratios in either a pure Ar or an N_2_ atmosphere (see samples 9–11 in Table 1 ▸). The largest fraction of Al_5_C_3_N phase was obtained in a sample with a short dwelling time at high temperature in an Ar atmosphere which confirms the fast solid-state reaction between Al_4_C_3_ and AlN. A molar ratio of 2:1 was used to compensate for the partial decomposition of Al_4_C_3_ at high temperatures (sample 9). Al_5_C_3_N is also formed from mixtures rich in AlN but in low yields (sample 10) which suggests that the graphitic carbon that forms from the decomposition of Al_4_C_3_ inhibits the solid-state reaction. The mixture heated in N_2_ shows that all Al_4_C_3_ reacts after two hours and the observed AlN phase has formed from the nitridation of Al.

The reaction pathways to form Al_5_C_3_N can thus be described as a fast gas–solid phase reaction involving the nitridation of Al_4_C_3_ or a solid-state reaction of Al_4_C_3_ with AlN depending on the reactants used. However, the latter reaction results in samples with both Al_5_C_3_N and Al_6_C_3_N_2_ phases due to the further reaction between Al_5_C_3_N and AlN. All characterizations discussed below are performed on samples synthesized using route (i).

### 3.2. Single-crystal X-ray diffraction of Al_5_C_3_N

Following route (i) above for the synthesis of a polycrystalline sample, we were able to isolate a few single crystals large enough for a structure determination using a single-crystal diffractometer. As previously described, in 1963, Jeffrey and Wu suggested that Al_5_C_3_N crystallized in noncentrosymmetric *P*6_3_*mc* space group. This structure, from a crystallographic point of view, is very similar to a structure in the centrosymmetric *P*6_3_/*mmc* space group. The difference between them is that due to the lack of inversion symmetry in *P*6_3_*mc*, N atoms can fully occupy either of two fourfold sites resulting in an ordered arrangement, whereas in *P*6_3_/*mmc* these sites are equivalent and partially occupied by C and N atoms. A final assignment of the structure to one of them depends on the quality of the diffraction data, and Jeffrey & Wu (1966 ▸) reported relatively high *R* factors, suggesting that the proposed *P*6_3_*mc* space group may be incorrect. To verify the space group symmetry, a small single crystal was selected and analyzed, and the space group test with the program *JANA2020* suggested three equally valid space groups *P*6_3_/*mmc* (#194), *P*62*c* (#190) and *P*6_3_*mc* (#186).

**Figure 2:**
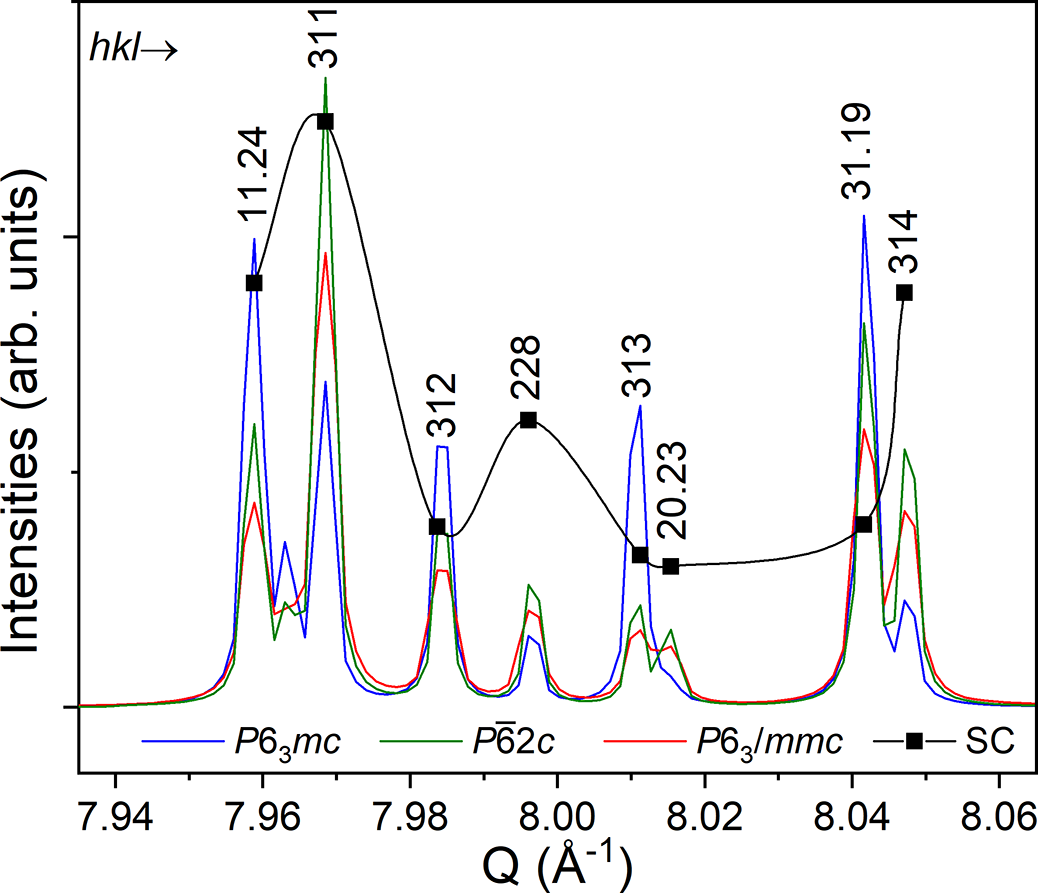
Comparison of the observed single-crystal peak intensities with the calculated intensities from the three structural models in space groups *P*6_3_*mc* (#186), *P*62*c* (#190) and *P*6_3_/*mmc* (#194).

The ordered structure model in *P*6_3_*mc* displays the expected layered structure with N in fourfold coordination as shown in Fig. 1 ▸. However, the structure solution in this space group did not converge during refinement and resulted in an atomic displacement for Al at (0, 0, ¼), *i.e.* at the equatorial position of the trigonal bipyramid, about 10 times higher than the average value at other Al positions. Alternatively, in space groups *P*6_3_/*mmc* and *P*62*c*, this Al site has to be described as a split site with half occupancy. In the refinement, N and C atoms were located at the same 4*f* Wyckoff position (⅓, ⅔, *z*) but residual electron density in the Fourier maps suggested that N also substituted for C at the 2*b* Wyckoff position (0, 0, ¼). A comparison of these possible models and well resolved peak intensities at high-*Q* values (Fig. 2 ▸) shows that the *P*6_3_*mc* model is not in agreement with the diffraction data from our crystal. A comparison of the proposed structural models and the well resolved peak intensities at high-*Q* values is presented in Fig. 2 ▸. As shown, the calculated intensity evolution for the *P*6_3_*mc* model deviates from the experimentally observed intensity trend, indicating that the *P*6_3_*mc* structure is not consistent with the diffraction data obtained from our crystal. Thus, the unstable refinement and large thermal displacement, see Table 2 ▸, suggest that the generally accepted space group *P*6_3_*mc* must be rejected. Moreover, the positive Flack parameter of +0.2 (5) for the *P*6_3_*mc* structure model indicates a possible inversion twin component. The choice between *P*62*c* and *P*6_3_/*mmc* was made in favor of the higher-symmetry model. The refinement in lower-symmetry space group *P*62*c* did not lead to a significant improvement in the agreement factors. Subsequently a joint refinement was carried out using SCXRD and neutron powder data in centrosymmetric space group *P*6_3_/*mmc*, see below. Parameters from the final refinements can be found in Table 2 ▸.

### 3.3. Neutron powder diffraction of Al_5_C_3_N

**Figure 3:**
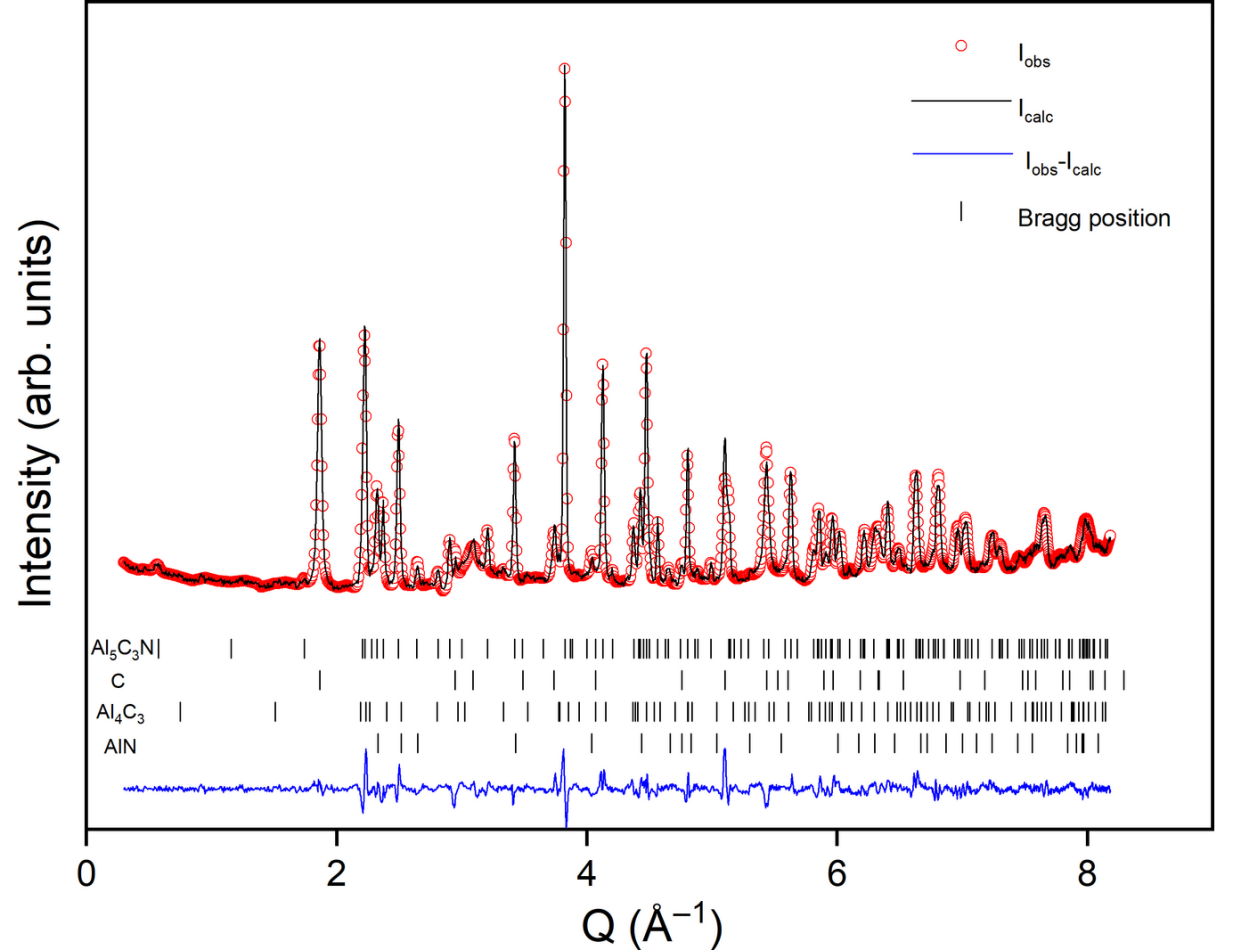
Results from Rietveld analysis of room-temperature neutron powder diffraction data (λ = 1.46 Å) for Al_5_C_3_N in *P*6_3_/*mmc*. Total χ^2^ = 6.9%, *R*_wp_ = 4.5%, Al_5_C_3_N phase *R*_Bragg_ = 5.5%. Phase fractions: Al_5_C_3_N 69.8%, C 24.5%, Al_4_C_3_ 3.5%, AlN 2.2%.

**Figure 4:**
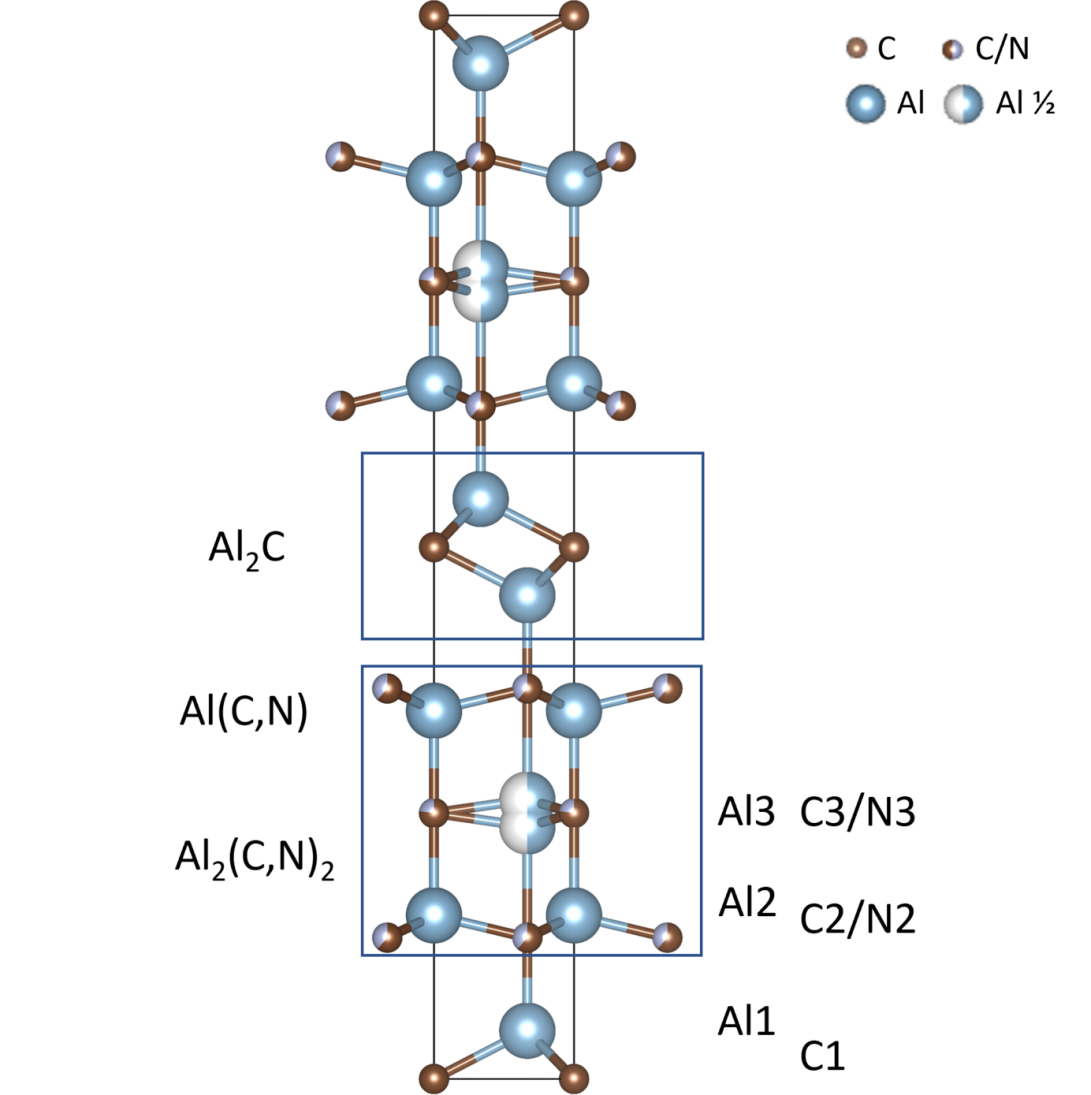
Structure model of Al_5_C_3_N in the *P*6_3_/*mmc* space group based on our data. The figure also shows the alternating slabs of Al_2_C–(AlN–Al_2_C_2_)– that in this space group are disordered due to partial occupancies.

The difference in neutron scattering length for C and N allows for the determination of their occupancies with less ambiguity. As observed during the single-crystal structure solution, in the preliminary refinements of the structural model of Al_5_C_3_N in the *P*6_3_*mc* space group, the position and displacement parameter for Al at *z* ≃ 0.25 were unstable and the disordered structure model in *P*6_3_/*mmc* was tested. Rietveld analyses resulted in similar models with N substituting partially at one or two C sites and in order to determine the best solution, a joint refinement with SCXRD data was done. The final model with a split Al site takes into account the residual electron density that was found in the Fourier maps and shows that N partially occupies two sites. The unit-cell parameters are *a* = 3.2829 (5), *c* = 21.604 (5) Å and *V* = 201.64 (6) Å^3^, and the final fit from the Rietveld refinement of the powder diffraction data is shown in Fig. 3 ▸. The structural model of Al_5_C_3_N in the *P*6_3_/*mmc* space group based on the joint refinement of the SCXRD and neutron diffraction data, Table 3 ▸, is shown in Fig. 4 ▸ and calculated distances are shown in Fig. S3.

### 3.4. Possible structural models of Al_5_C_3_N

Based on the results from the single crystal X-ray and neutron powder diffraction studies, the structure is better described as a disordered structure in the centrosymmetric *P*6_3_/*mmc* space group. This disorder has been modeled in similar compounds with inversion twins, *i.e.* with the combination of two noncentrosymmetric structures related by an inversion center in an alternative structure solution based on an inversion twin. This type of twinning would not be evident from diffraction data as there are no significant differences in the calculated peak intensities between the structural models. However, an inversion center can be confirmed by measurements of physicochemical properties (Ok *et al.*, 2026 ▸) or measurements of the active modes in Raman and IR spectra (see Fig. S5). Austerman & Gehman (1966 ▸) have shown that such twins can be created in wurtzite-type crystals during crystal growth with impurities or crystal strains, *e.g.* oxygen in AlN or strain gradients in BeO. A similar solution has been proposed for the structure of Al_3_BC from X-ray diffraction data (Meyer & Hillebrecht, 1997 ▸), with a centrosymmetric model and the equatorial Al position of the trigonal bipyramid split along the *c* axis. The origin of the static disorder was attributed to twinning and a later computational study proposed a structure modeling this disorder (Huguenot *et al.*, 2020 ▸). In these cases, models with split positions are the average structure as observed in a polycrystalline material but the split site can also be described using a propagation vector in a lower symmetry orthorhombic space group that simulates the movement of this atom.

**Figure 5:**
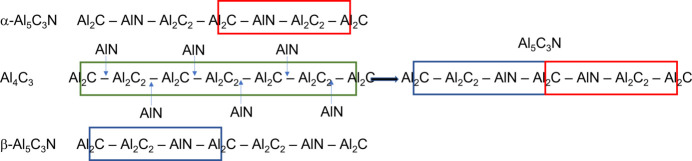
Models for the formation of inversion twins in the Al_5_C_3_N structure formed from the insertion of AlN in Al_4_C_3_ (left) and the Al_5_C_3_N twin formed by a combination of α-Al_5_C_3_N and β-Al_5_C_3_N (right).

A possible pathway to the formation of such inversion twins is outlined in Fig. 5 ▸. Al_4_C_3_ can be described as a layered structure consisting of alternating Al_2_C and Al_2_C_2_ units. It is possible to form the Al_5_C_3_N phase by inserting AlN layers between these units in two ways as illustrated in Fig. 5 ▸. The stacking sequence can then be either –Al_2_C–AlN–Al_2_C_2_– (denoted α-Al_5_C_3_N) or –Al_2_C_2_–AlN–Al_2_C– (denoted β-Al_5_C_3_N). The α-Al_5_C_3_N sequence is, in fact, the noncentrosymmetric *P*6_3_*mc* crystal structure of Jeffrey & Wu (1963 ▸) and even though α-Al_5_C_3_N and β-Al_5_C_3_N are separately noncentrosymmetric, they are related by inversion symmetry. The combination of both stacking sequences will produce a pseudo-symmetric structure which would be identified as *P*6_3_/*mmc* in our X-ray and neutron diffraction studies above. In each case, an Al_2_C–Al_2_C_2_ unit separates the AlN layers, keeping two AlN layers as far away from each other as possible and ensuring that the AlN layers are maximally dispersed.

However, a potential problem with the twinned structure outlined in Fig. 5 ▸ is the formation of a twin boundary at an energy cost. A second possibility is that both α-Al_5_C_3_N and β-Al_5_C_3_N can coexist as a locally disordered phase within a supercell. This disorder generally makes the structure energetically less favorable unless the disorder gives rise to a periodic lattice distortion that extends beyond the unit cell, akin to a charge-density wave, which will lower the energy of the overall structure and induce a spontaneous symmetry breaking. In the next section, the stability of these two possibilities is investigated using DFT.

### 3.5. Theoretical calculations

**Figure 6:**
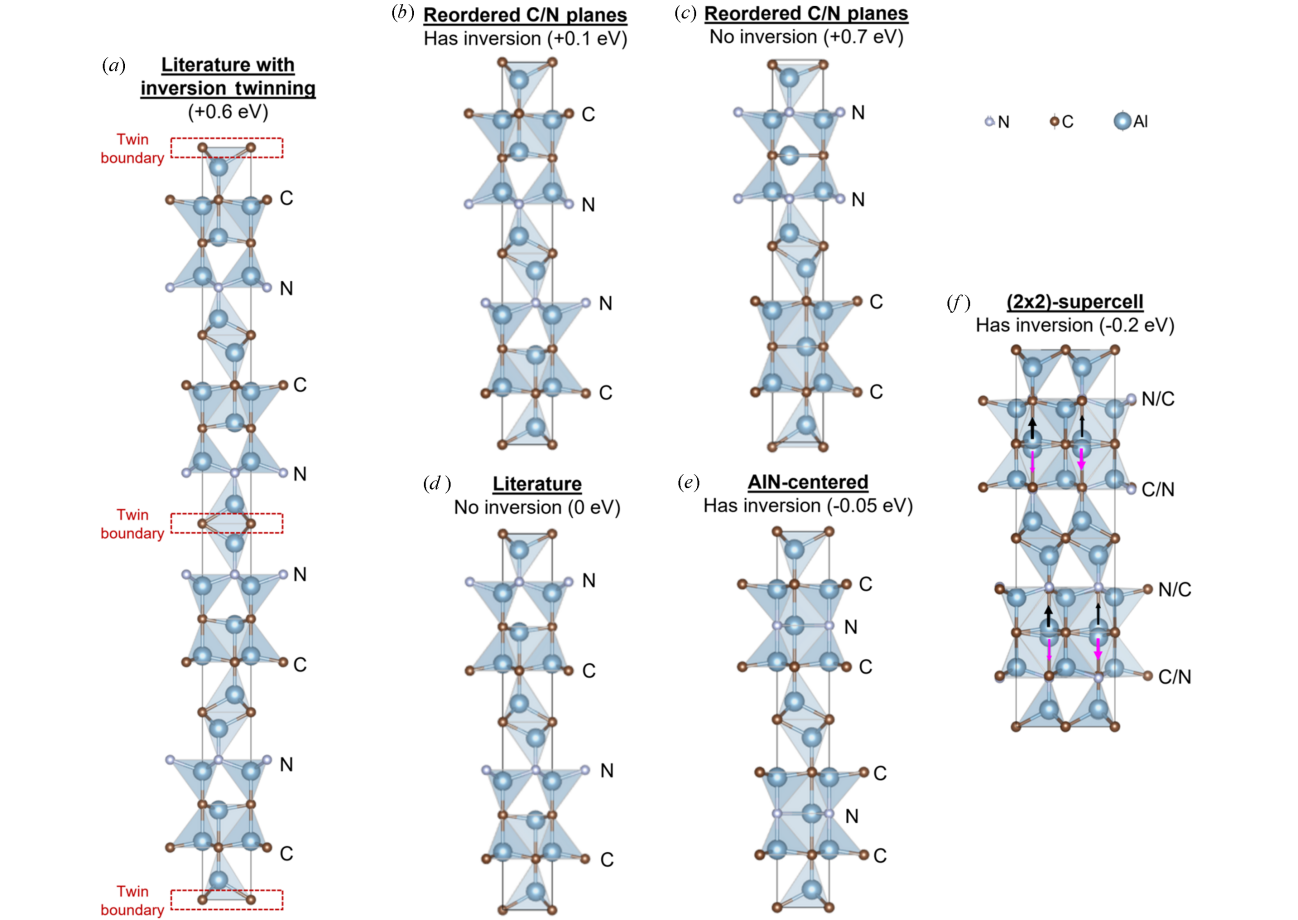
Candidate structures of Al_5_C_3_N and their formation energies per f.u. relative to the published *P*6_3_*mc* structure of Jeffrey & Wu (1963 ▸) as shown in (*d*), which contains two f.u. (*a*) shows the published structure twinned along the *c* axis and its energetically unfavorable twin boundaries. (*b*) and (*c*) show the published structure with two other possible site occupancies for N related by inversion symmetry in (*b*) and or not in (*c*). Both (*b*) and (*c*) are energetically less favorable than the published structure in (*d*), which has its AlN planes maximally separated. (*e*) shows the Jeffrey & Wu (1963 ▸) structure with an AlN plane centered in the middle of the slab, (*f*) shows our proposed structure, which restores the inversion symmetry via the even atomic distributions of C/N within the same plane. This structure has the lowest energy due to spontaneous symmetry breaking within the (2×2)-supercell. The magenta/black arrows denote the downwards/upwards displacements of Al due to modulations in the local chemical environment.

In order to correctly describe the structure of the Al_5_C_3_N phase and to understand the origin of the inversion symmetry, we begin by calculating the DFT energy of the *P*6_3_*mc* structure (Jeffrey & Wu, 1963 ▸) [denoted as ‘Literature’ in Fig. 6 ▸(*d*)]. First, we explore the possibility that the inversion symmetry that was determined from the diffraction experiments arises from naturally occurring inversion twins at the energetic cost of forming twin boundaries. We investigated all possible periodic positions of the twin boundary along the *c* axis and found that when the twin boundary is located as shown in Fig. 6 ▸(*a*), each boundary would have the lowest formation energy of 0.6 eV per unit cell in the *ab* plane, which is still prohibitively large and unlikely to be formed experimentally. In this work, the formation energy is defined as the DFT-calculated total energy of the configuration minus the DFT energy of the structure reported in the literature [see Fig. 6 ▸(*d*)].

We then calculate the formation energy needed to reorder the C/N planes within the unit cell, with respect to the *P*6_3_*mc* structure. All permutations are considered and we will first discuss the two configurations [Figs. 6 ▸(*b*) and 6 ▸(*c*)] where each AlN plane is formed along the surface of the Al_2_C slabs. In the first configuration [Fig. 6 ▸(*b*)], we restore the inversion symmetry by ordering the AlN planes such that they become related by inversion symmetry. In this configuration, the two N planes are closer to each other than in the *P*6_3_*mc* structure. This configuration has a positive formation energy per Al_5_C_3_N formula unit (f.u.) of 0.1 eV, suggesting that it is energetically more favorable to keep the AlN planes apart. We hypothesize that this is because N has a lower electron affinity than C, and is unable to accept all the electrons that Al would like to donate in order to maintain charge neutrality. This would lead to the n-type doping of Al and the p-type doping of C. The lack of a complete shell leads to higher energy because it lacks the exchange-correlation stabilization and minimized repulsion of a closed-shell configuration. The system is thus more reactive and becomes less stable.

**Figure 7:**
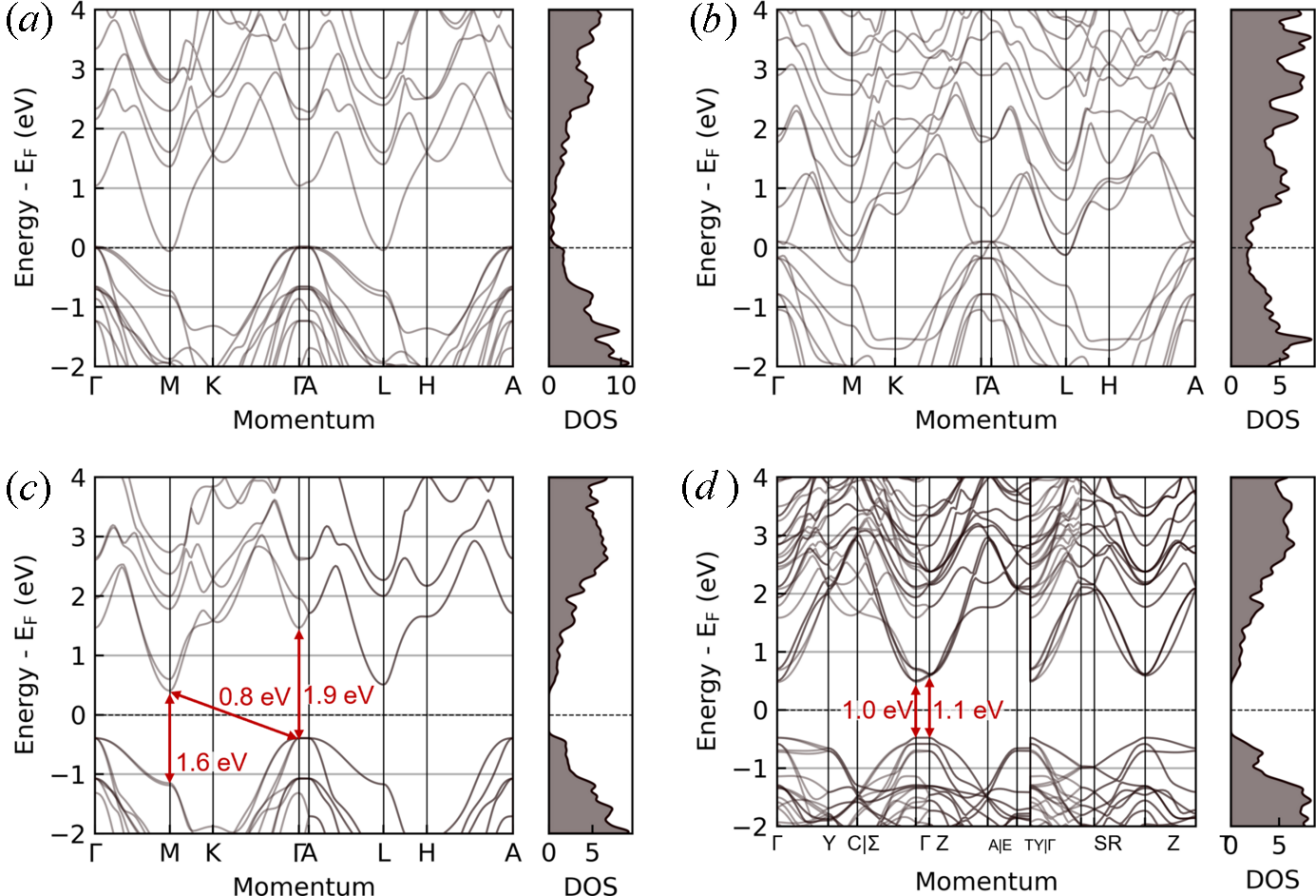
(*a*)–(*d*) Band structures of the structures shown in Figs. 6 ▸(*b*), 6 ▸(*c*), 6 ▸(*d*) and 6 ▸(*f*), respectively. Each band structure is accompanied by the density of states (DOS) on the right that is normalized to the number of atoms within the unit cell of Al_5_C_3_N.

To verify our hypothesis, we calculated the band structure of this configuration in Fig. 7 ▸(*a*) and compared it against that of the *P*6_3_*mc* structure in Fig. 6 ▸(*b*). Indeed, we found states at the Fermi level due to p and n-types doping for the more unstable structure. We further test our hypothesis by re­ordering the AlN planes in a second configuration [Fig. 6 ▸(*c*)], such that both the AlN planes are now even closer. Not only does this configuration not have an inversion center, the formation energy is even larger than the first configuration (0.7 eV per f.u.), and the p-/n-types doping becomes more severe [Fig. 7 ▸(*b*)]. Having established that the AlN planes prefer to be located as far away from each other as possible, we question why the AlN planes would preferentially locate along one of the two surfaces of the –Al_2_C–Al_2_C_2_– slabs, as suggested by Jeffrey & Wu (1966 ▸). If each AlN-plane is located in the middle of the slab, not only would the overall structure have a higher symmetry belonging to the space group *P*6_3_/*mmc*, this structure will also have an inversion symmetry and the space group observed in the X-ray diffraction experiments. In fact, our calculations show that such a structure would even have a negative formation energy of −0.05 eV/f.u. [Fig. 6 ▸(*e*)] compared to the reference structure [Fig. 6 ▸(*d*)], and is, therefore, more stable than the *P*6_3_*mc* structure proposed by Jeffrey & Wu (1963 ▸), Jeffrey & Wu (1966 ▸). Nonetheless, our diffraction experiments also confirm that this hypothetical ordered structure is not a desirable solution.

Finally, we consider the possibility in which lattice modulation extends the periodicity beyond that of the unit cell along the *ab* plane. This can induce spontaneous symmetry breaking that lowers the energy of the overall structure, akin to a charge-density wave. To this end, we created a (2×2)-supercell with even atomic distributions of C/N within the same plane [Fig. 6 ▸(*f*)] and of all the structures we have evaluated, this structure has the lowest energy, that is 0.2 eV/f.u. lower than the published structure (Jeffrey & Wu, 1963 ▸). The formation energy of the structure is not only negative, confirming its thermodynamic favorability, but is also large in magnitude. In Fig. 6 ▸(*f*), we see that the checkered atomic distribution of C and N creates modulations in the local chemical environment that cause Al in the now Al_2_CN slab to be displaced slightly upward (black arrows) and downward (magenta arrows) in the *c* direction. Such atomic displacements are confirmed experimentally in our diffraction studies as a split position, and were also observed for Al_4_SiC_4_ (Ong *et al.*, 2024 ▸), for which we also reassessed its crystal symmetry. Summarizing, inversion symmetry can be restored via the fractional occupation of Al(C,N) layers [Fig. 6 ▸(*f*)]. Such a structure can be interpreted as a disordered superposition of the (1×1)-unit cells that individually do not have inversion symmetry, as shown in Fig. 6 ▸(*d*). Indeed, the experimental results indicate additional disorder with N substituting at the two sites indicated in Figs. 6 ▸(*e*) and 6 ▸(*f*), both energetically more favorable than the literature structure in Fig. 6 ▸(*d*). However, due to computational constrains, the modeling of a structure with this degree of disorder was not carried out.

In Fig. 7 ▸, we show the calculated electronic structure of the structures considered for the Al_5_C_3_N compound. Note that depending on crystal structure, the electronic structure is semi-conducting, semi-metallic or metallic. Fig. 7 ▸(*c*) shows also the calculated DFT bandgap and we see that the *P*6_3_*mc* structure has a direct gap of (1.6–1.9 eV) and an indirect gap of 0.8 eV, in agreement with Xu *et al.* (2011 ▸). In the 2×2 supercell, Fig. 7 ▸(*d*), the conduction bands were folded into the Γ point, and the breaking of discrete translational symmetry has allowed for direct transition of 1.0 eV at the Γ point. Nonetheless, we note that since DFT bandgaps are not quasiparticle bandgaps, they underestimate the experimental bandgap of 2.2 eV, as expected from calculations of DFT level (Gai *et al.*, 2025 ▸).

### 3.6. STEM

**Figure 8:**
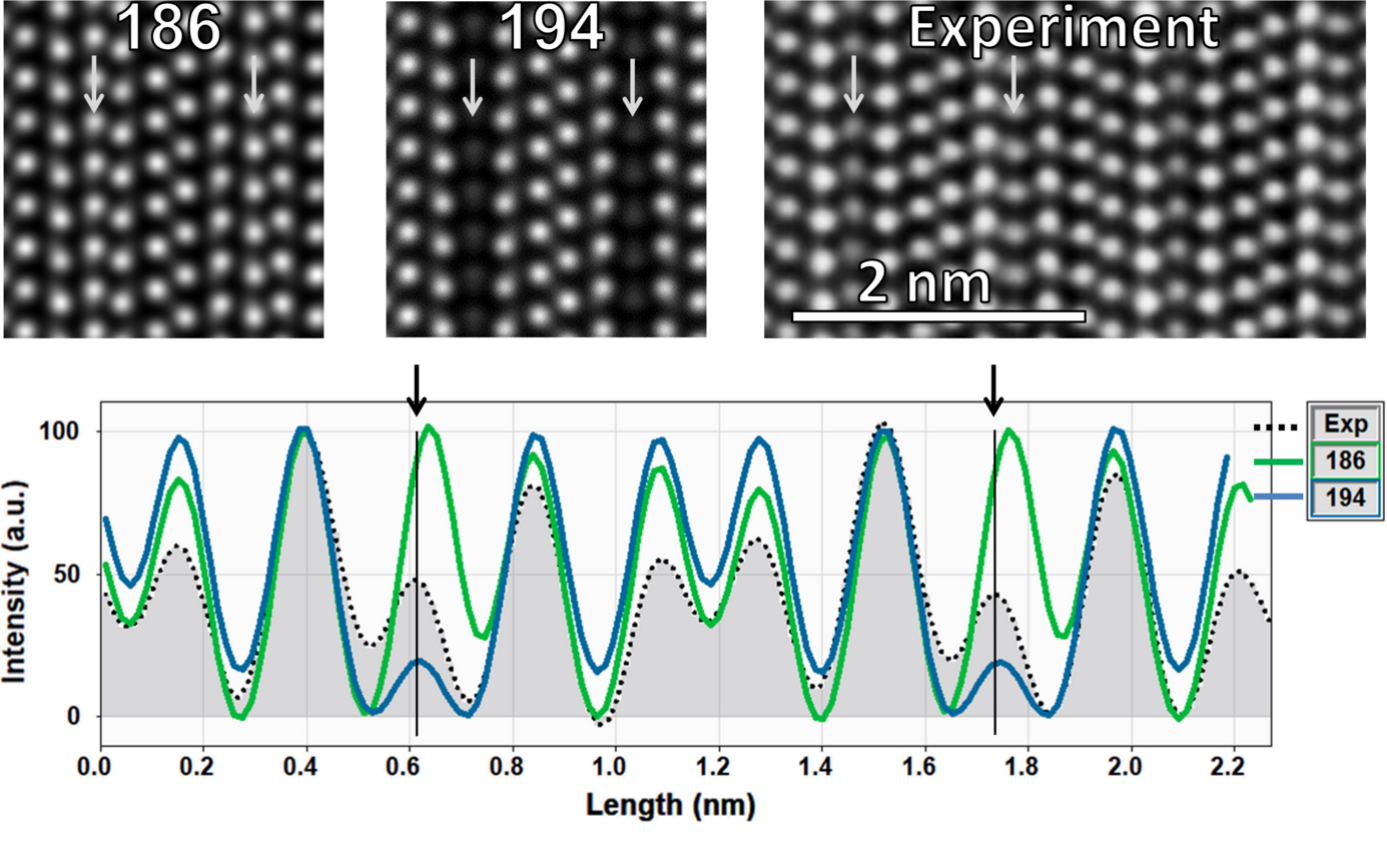
(top) Simulated HAADF STEM images from the model with space groups *P*6_3_*mc* (#186) and *P*6_3_/*mmc* (#194) using *Dr. Probe* software and a frozen lattice procedure (50 frozen states and total thickness of 20 nm) compared to the experimental one. (bottom) Compilation of the horizontal line profiles of the HAADF intensities integrated over the height of the image. The vertical arrows in the intensity profiles and in the HAADF images point to the same double-Al atomic plane. For an easier comparison, intensity curves were background corrected and normalized at the Al(C,N) atomic plane (close to 0.4 nm).

Our experimental results as well as the DFT calculations in Section 3.5 clearly suggest that the generally accepted crystal structure for Al_5_C_3_N with an ordered and noncentrosymmetric *P*6_3_*mc* (#186) space group is not correct, and that a centrosymmetric and disordered structure in *P*6_3_*/mmc* (#194) is a better fit to our results. To confirm this conclusion, a STEM study was carried out on crystals of Al_5_C_3_N. Experimental and simulated HAADF STEM images are shown in Fig. 8 ▸.

While the models *P*6_3_*mc* (#186) and *P*6_3_/*mmc* (#194) are closely related, some of the atomic plane spacings are slightly different due to the splitting of the Al site in *P*6_3_/*mmc*. Deviations for model *P*6_3_*mc* are evident in Fig. 8 ▸ (bottom) as indicated by the vertical lines at about 0.6 and 1.75 nm corresponding to the split Al sites, where *P*6_3_/*mmc* is closer to the experiment. Concerning the change in HAADF intensities, one can see that none of the current lattices perfectly fit the experiment, but once again the model in *P*6_3_/*mmc* is closer, with intensities being higher at the Al(C,N) planes and lower at the split Al ones. The intensity at the central Al_2_C plane also decreases for our model however not enough to match the experimental results. In other words, the apparent density of these atomic planes does not reflect exactly the STEM experiment and could indicate a different amount of vacancy as discussed in a previous work for Al_4_SiC_4_ (Ong *et al.*, 2024 ▸).

**Figure 9:**
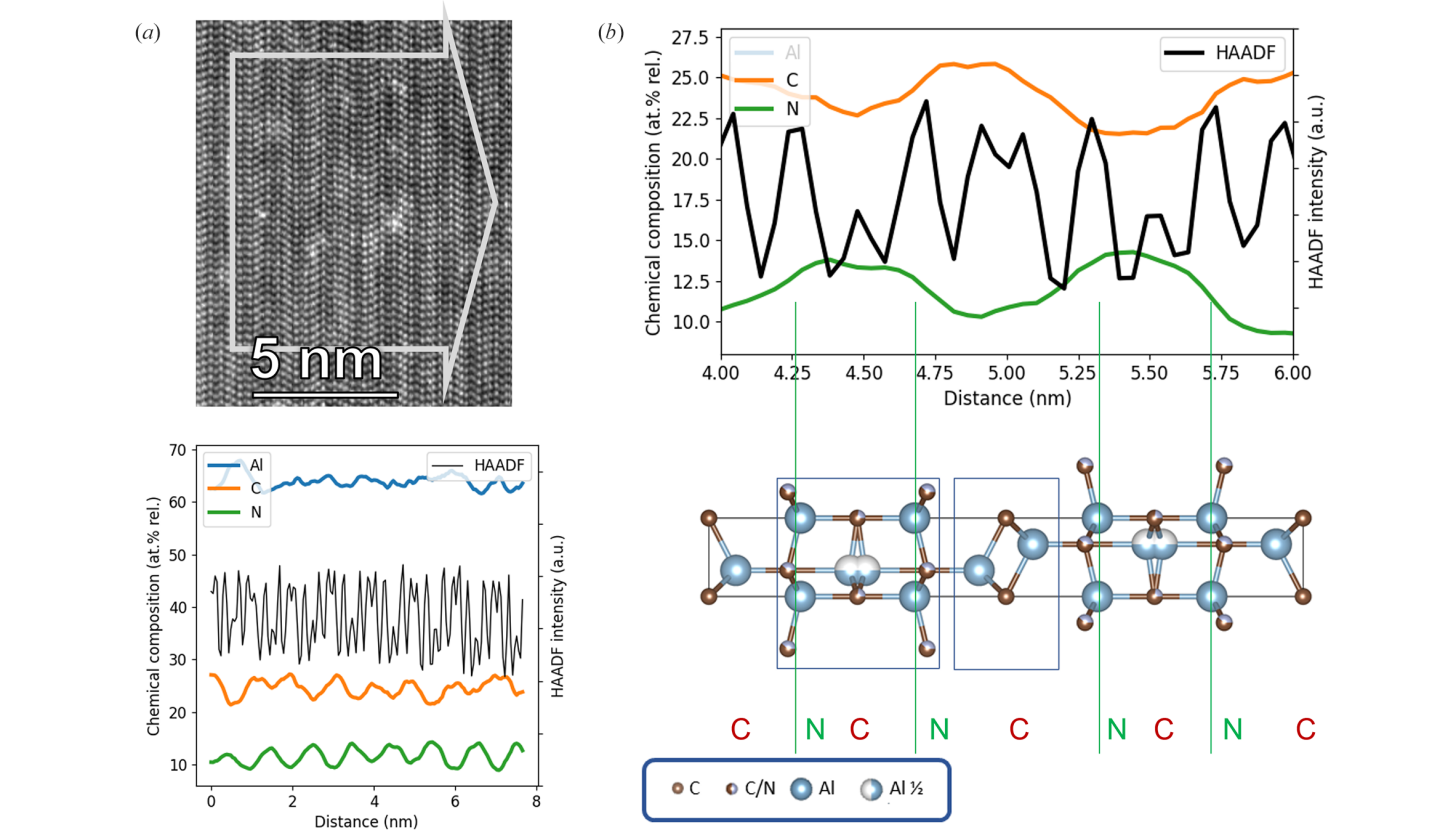
(*a*) (top) STEM HAADF survey image of the EDS mapped region, bottom, the corresponding integrated profiles of the chemical composition showing repetitive patterns for Al and N signals. (*b*) (top) Cropped integrated profiles to compare it to our atomic model as presented in Fig. 4 ▸. EDS measured in one single scan; the signal is integrated over a width of 10 nm as indicated by the gray arrow on the HAADF survey image.

The analysis of the chemical composition using EDS is presented Fig. 9 ▸. The HAADF profile shows a similar sequence to the high-resolution scanning electron microscopy measurements in Fig. 8 ▸ despite of the lower resolution due to the decreased sampling rate and larger electron probe utilized to get a useful count rate for the chemical mapping. In spite of the low X-ray counts, both integrated profiles for C and N feature clear repetitive sequences that can be correlated to the atomic model *P*6_3_/*mmc* (#194). Indeed, N is localized at Al(C,N) planes and C is localized at Al_2_C planes. The fact that the quantified amounts of neither C nor N do not fall to zero even where it would be expected from the model is not surprising and can be explained by an insufficient spatial resolution. First, the higher beam current used for the measurement (∼230 pA) translates as a larger probe size which enhances the EDS signal but degrades the spatial resolution as the tail of the Gaussian probe would always slightly excite neighboring atomic planes. Second, binning of the recorded data was used to enhance the signal-to-noise ratio which also broadens the spatial resolution. The thickness of the lamella has also been reported to rapidly reduce the chemical composition spatial resolution by broadening signals from one atomic plane to neighboring ones. As a consequence, the peak maxima (and valley minima) are weighted by neighboring pixels and lose amplitude, and the detectability criterion as described by Lu *et al.* (2014 ▸) may not be fulfilled for the shorter interatomic distances, insufficient to resolve each planes properly. However, by accepting a lower signal-to-noise ratio and decreasing the binning level, the C signal start to emerge at several Al split sites as presented in Fig. S6, supporting further the refined model presented in this work.

## 4. Related literature

The following reference is cited only in the supporting information: Pedesseau *et al.* (2017 ▸).

## 5. Conclusions

We have thoroughly revisited the crystal structure of the Al_5_C_3_N compound. Based on the variety of experimental and theoretical techniques employed in this work, we conclude that Al_5_C_3_N samples can be prepared from Al_4_C_3_ at high temperatures and N_2_ partial pressures close to 1%. Al_5_C_3_N crystallizes in a centrosymmetric space group [*P*6_3_/*mmc* (#194)] rather than the previously reported noncentrosymmetric one [*P*6_3_*mc* (#186)] with the following observations supporting this conclusion.

(i) SCXRD shows that the variation of observed peak intensities follows the same trend as expected for the centrosymmetric space group, while refinements in the noncentrosymmetric space group do not converge.

(ii) Joint refinement of SCXRD and neutron powder diffraction data indicates that N and C are disordered at the 4*f* and 2*b* Wyckoff sites.

(iii) The possibility of inversion twinning, which could produce a pseudo-symmetric structure, would be energetically unfavorable.

(iv) DFT calculations also demonstrate that a centrosymmetric structure has the lowest formation energy but it was not feasible to perform calculations on a structure with N disordered over two sites.

(v) STEM results reveal a variation in intensities consistent with SCXRD, confirming that the crystal structure of Al_5_C_3_N is disordered. STEM HAADF combined with EDS further shows C and N compositional variation, in agreement with the diffraction results.

Our findings are expected to support future investigations of layered structures in the Al–C–N and Al–C–Si–N systems. The revised disordered crystal structure of the Al_5_C_3_N compound will be crucial for theoretical predictions of its physical properties.

## Supplementary Material

Crystal structure: contains datablock(s) global, I. DOI: 10.1107/S2052520626002544/ra5167sup1.cif

Figs. S1-S6, Table S1. DOI: 10.1107/S2052520626002544/ra5167sup2.pdf

CCDC reference: 2504685

## Figures and Tables

**Table 1:** Phase fractions determined using the reference intensity ratio method from powder diffractograms in samples heated once at different temperatures and partial pressures of N_2_ The numbers in parentheses for sample 7 indicate the fractions obtained after a second sintering.

					Phase fractions (wt%)				
Sample	Atmosphere	Al_4_C_3_:AlN	*T* (K)	*t* (min)	Al_5_C_3_N	Al_6_C_3_N_2_	Al_4_C_3_	AlN	Reactions†
1	Ar	1:0	2173	60			100		(1)
2	Ar	1:0	2273	60			100		(1)
3	N_2_	1:0	2173	60			30	70	(1), (2)
4	N_2_	1:0	2273	10	5		90	5	(1), (2), (4)
5	10% N_2_	1:0	2173	60	40	5	35	20	(1), (2), (4), (6)
6	4% N_2_	1:0	2273	60	60		20	20	(1), (4), (5)
7	1.5% N_2_	1:0	2273	30	20 (70)		80 (30)		(1), (4)
8	1% N_2_	1:0	2273	120	40		60		(1), (4)
9	Ar	2:1	2273	10‡	70		10	20	(1), (3)
10	Ar	1:4	2273	60	20			80	(1), (3)
11	N_2_	1:1	2273	120	50	5		45	(1), (2), (3), (4), (6)

† (1) Al_4_C_3_ → 4Al + 3C; (2) Al + ½ N_2_ → AlN; (3) Al_4_C_3_ + AlN → Al_5_C_3_N; (4) 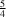
Al_4_C_3_ + ½N_2_ (g) → Al_5_C_3_N + 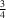
C; (5) Al_5_C_3_N + 2N_2_ (g) → 5AlN + 3C; (6) Al_5_C_3_N + AlN → Al_6_C_3_N_2_.

‡ Sample heated at about 65 K min^−1^.

**Table 2:** Results of the crystal structure refinements of Al_5_C_3_N in different space groups based on the SCXRD data

	*P*6_3_*mc* (#186)	*P*622*c* (#190)	*P*6_3_/*mmc* (#194)
Al3 site occupancy at ( 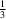 , 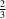 , *z*)	1.0 at 2*b*	0.5 at 4*f*	0.5 at 4*f*
Anisotropic displacement *U*_33_ for Al3 at 2*b* or 4*f* (Å^2^)	0.122 (4)	0.0119 (18)	0.0106 (15)
Flack parameter	0.2 (5)	–	–
No. of independent reflections	408 (*R*_eq_ = 0.0462)	296 (*R*_eq_ = 0.0468)	210 (*R*_eq_ = 0.0471)
No. of reflections with *I* > 2σ(*I*)	327 (*R*_σ_ = 0.0234)	240 (*R*_σ_ = 0.02)	138 (*R*_σ_ = 0.0589)
Data, refined parameters	408, 27	296, 16	210, 17
Goodness-of-fit on *F*^2^	2.98	1.72	1.16
Final *R* indices [*I* > 2σ(*I*)]	*R*_1_ = 0.0548, *wR*_2_ = 0.1417	*R*_1_ = 0.0312, *wR*_2_ = 0.0868	*R*_1_ = 0.0281, *wR*_2_ = 0.1013
*R* indices (all data)	*R*_1_ = 0.0692, *wR*_2_ = 0.1456	*R*_1_ = 0.0428, *wR*_2_ = 0.0900	*R*_1_ = 0.0444, *wR*_2_ = 0.1047
Largest diff. peak and hole (e Å^−3^)	0.59 and −0.46	0.33 and −0.36	0.30 and −0.24

**Table 3:** Refined atomic positions and temperature factors from joint refinement of the SCXRD and neutron powder diffraction data for Al_5_C_3_N in space group *P*6_3_/*mmc* (#194) The total N occupancy was restrained to the nominal stoichiometry.

Site	Wyckoff	*x*	*y*	*z*	*U*_iso_ (Å^2^)	Occupancy
Al1	4*f*	1/3	2/3	0.04537 (7)	0.0079 (8)	1
Al2	4*e*	0	0	0.15384 (8)	0.0091 (8)	1
Al3	4*f*	1/3	2/3	0.2373 (1)	0.0049 (8)	0.5
C1	2*a*	0	0	0	0.0064 (6)	1
*M*2	4*f*	1/3	2/3	0.1329 (2)	0.0087 (6)	0.61C + 0.39N
*M*3	2*b*	0	0	1/4	0.0061 (6)	0.77C + 0.23N
