# Supplementary material for: Unraveling the symmetry of Al5C3N
Source: Acta Crystallogr B Struct Sci Cryst Eng Mater. 2026 Apr 22;82(Pt 3):299–309. doi: 10.1107/S2052520626002544 (PMC13238491; doi:10.1107/S2052520626002544)
Supplement: Supplementary file 2 [file b-82-00299-sup2.pdf]

STRUCTURAL SCIENCE
CRYSTAL ENGINEERING
MATERIALS

Volume 82 (2026)

Supporting information for article:

Unraveling the symmetry of Al5C3N

Vitalii Shtender, Chin Shen Ong, Pedro Berastegui, Olivier Donzel-Gargand, Johan Cedervall, Charles Hervoches, Premek Beran, Olle Eriksson and Ulf Jansson

Supplementary information

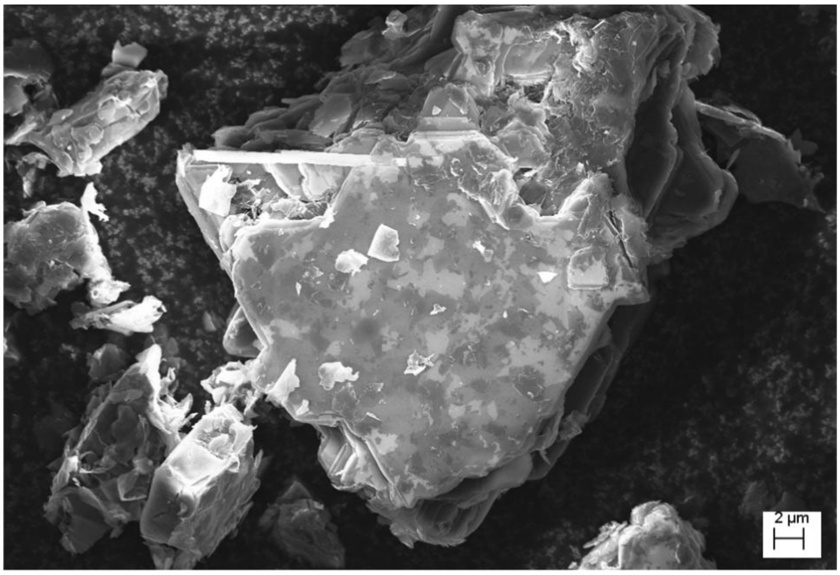

Figure SI1. *SEM image of an Al_5C_3N crystal.*

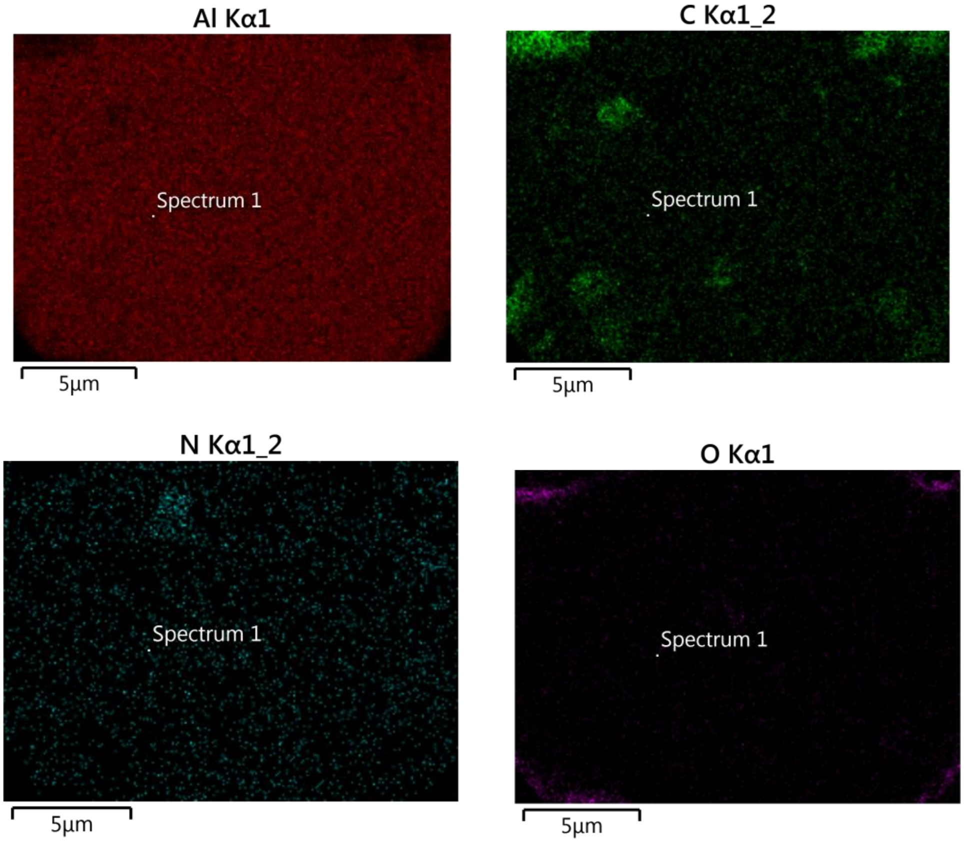

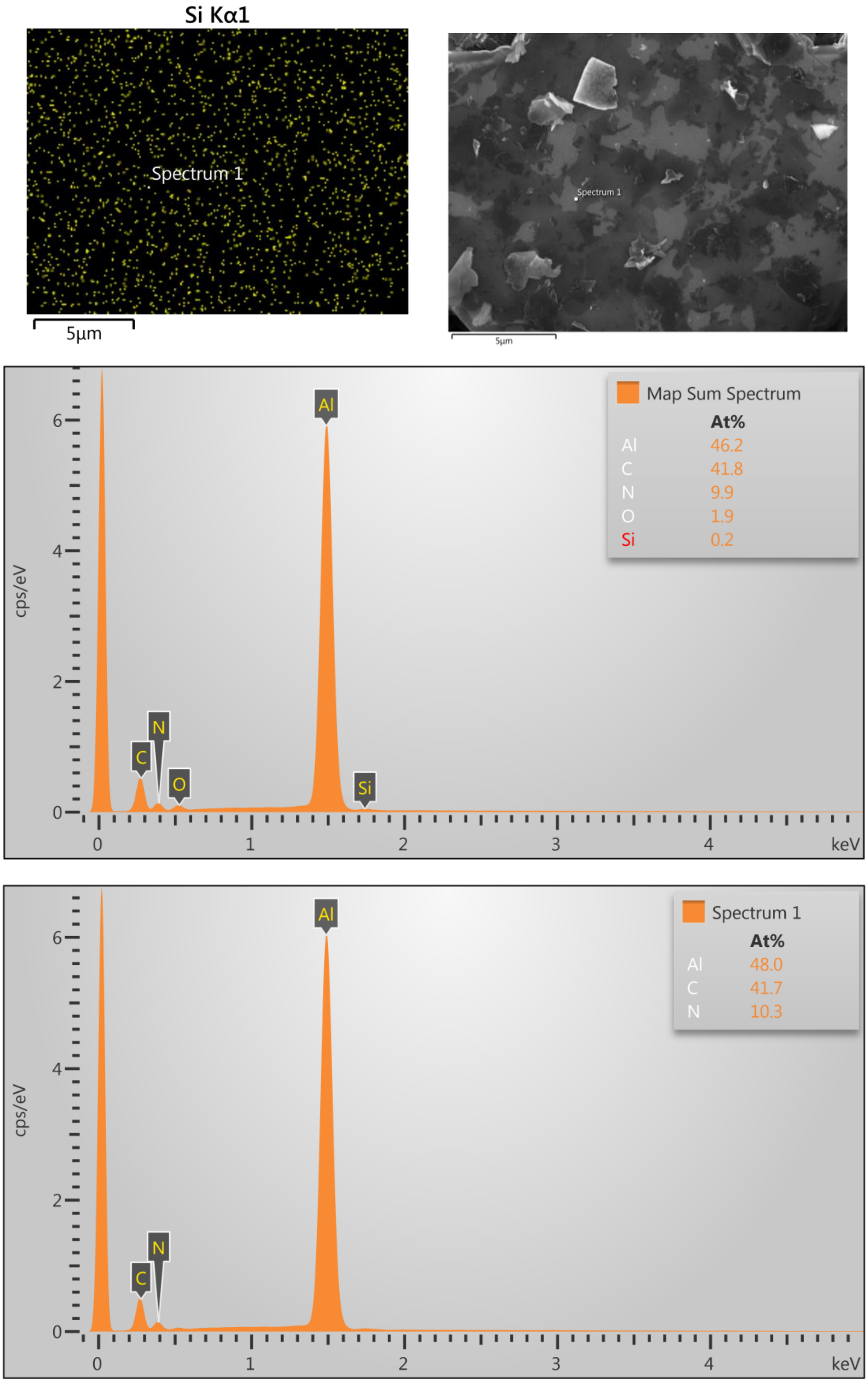

Figure SI2. *EDS analysis of an Al_5C_3N crystal. The measured oxygen content is very low and C impurities and an AlN flake are visible.*

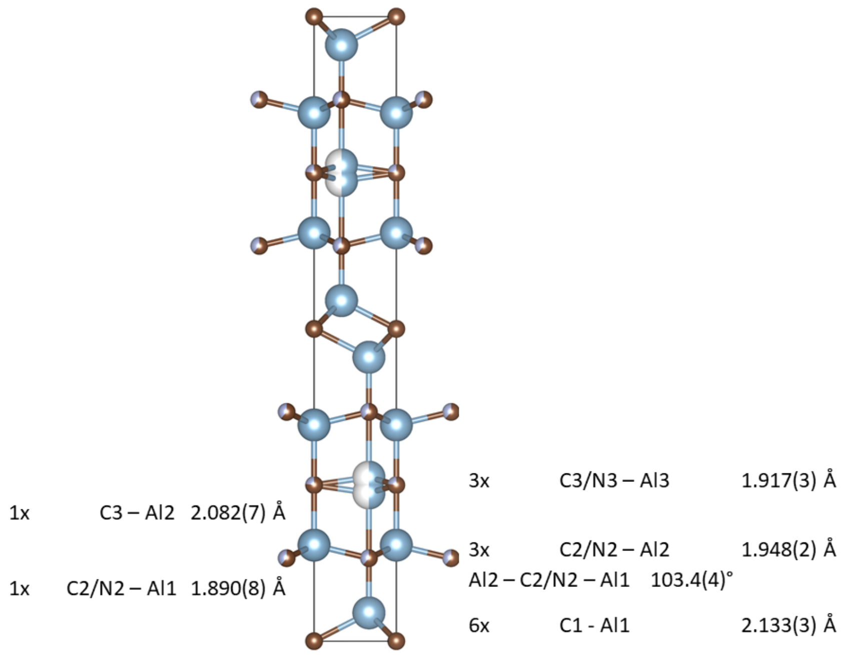

Figure SI3. *Bond distances in Å and tetrahedral angles calculated for the Al_5C_3N structure model in P6$_3$/mmc from neutron diffraction data. The bonding is illustrated for C sites (brown) and C/N sites (blue/brown) while Al sites (blue) are shown with larger spheres.*

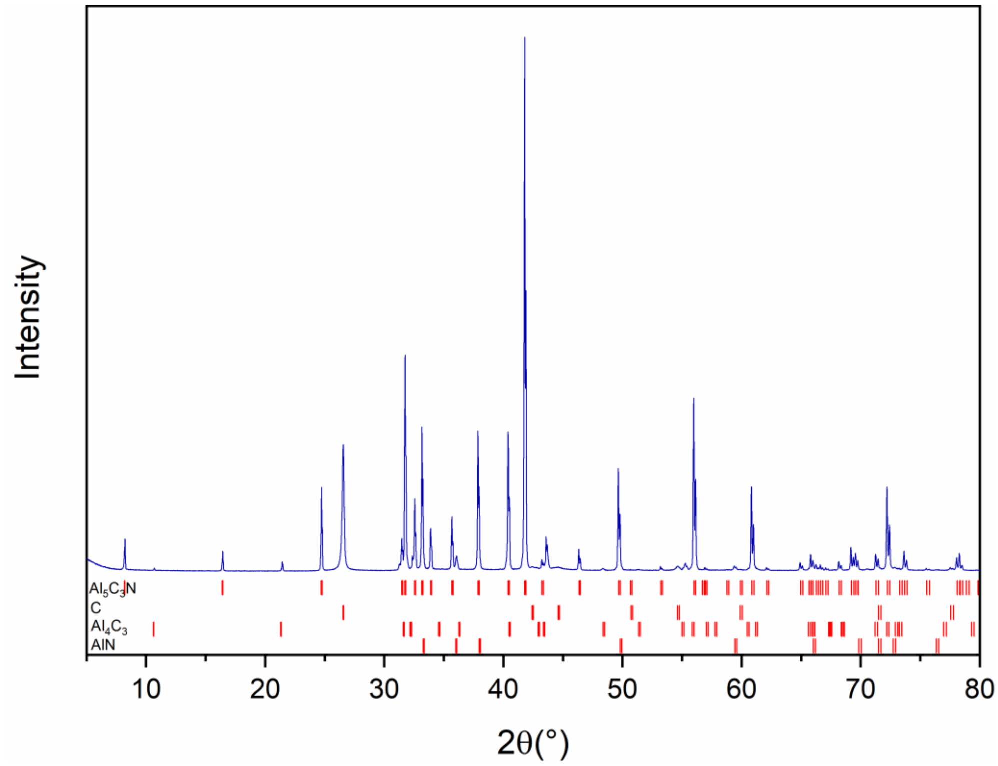

Figure SI4. *X-ray diffractogram of the Al_5C_3N sample with 2H-C as a second phase (peak at 26.6°) and Al_4C_3 and AlN as impurities. The main phase is Al_5C_3N with a= 3.2833(3) and c= 21.618(3) Å.*

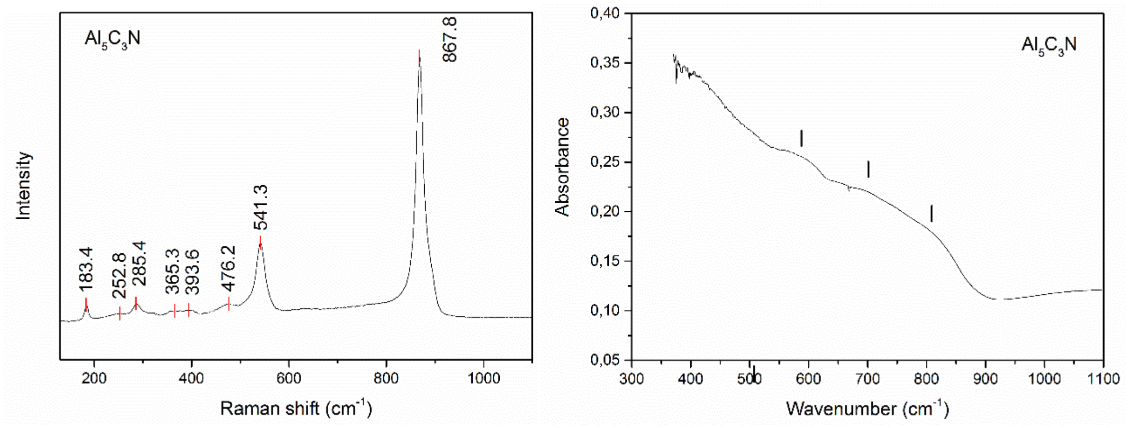

Figure SI5. Raman and ATR (attenuated total reflection) spectra from a polycrystalline Al_5C_3N sample. The Raman spectrum of Al_5C_3N shows strong A1 and E1 modes. The peak at 365 cm^{-1} could be assigned to a silent B2 mode and has been attributed to impurities (Pedesseau et al., 2017). The IR spectrum shows weak overlapping modes and the two strong A1 modes observed in the Raman spectrum (at 541 and 868 cm^{-1}) are not observed in the IR spectrum (520-630 and 775-875 cm^{-1}) which would indicate a centrosymmetric structure.

Table SI1. Experimental positions in cm^{-1} of Al_5C_3N Raman modes and IR (ATR: Attenuated Total Reflection, marked positions in the figure) modes.

ω_{exp} Raman	ω_{exp} ATR	Symmetry
183		E1
252		E2
285		E1
365		
393		A1
476		E1
541	590	A1
	740	E1
868	810	A1

L. Pedesseau, O. Chaix-Pluchery, M. Modreanu, *et al.*. *J Raman Spectrosc* 2017, **48**, 891-896.

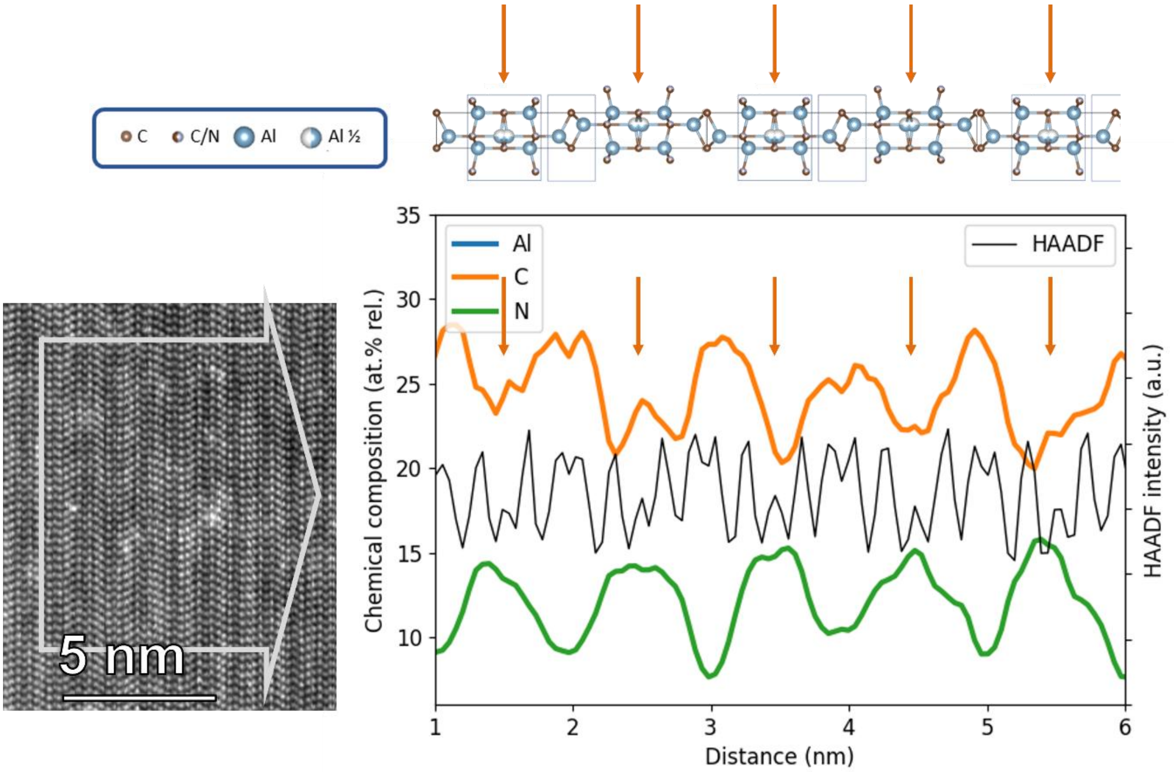

Figure SI6. *Identical dataset as presented in Fig. 9 but processed with a much lower binning rate (5 pixels instead of 11). The atomic model is scaled and aligned to the composition profile. When the binning is reduced, carbon signals starts to be visible in several Al(C,N) planes as indicated by the orange arrows. Nonetheless this signal is not totally consistent due to the higher signal to noise ratio.*
